# Vision-Based Hand Function Evaluation with Soft Robotic Rehabilitation Glove

**DOI:** 10.3390/s26010138

**Published:** 2025-12-25

**Authors:** Mukun Tong, Michael Cheung, Yixing Lei, Mauricio Villarroel, Liang He

**Affiliations:** 1 Department of Engineering Science, University of Oxford, Oxford OX1 3PJ, UK; mukun.tong@eng.ox.ac.uk (M.T.); michael.cheung@hmc.ox.ac.uk (M.C.); chri6867@ox.ac.uk (Y.L.); mauricio.villarroel@eng.ox.ac.uk (M.V.); 2 The Podium Institute for Sports Medicine and Technology, University of Oxford, Oxford OX1 3PJ, UK

**Keywords:** soft robotic glove, computer vision, hand pose estimation, hand rehabilitation, quantitative evaluation

## Abstract

Advances in robotic technology for hand rehabilitation, particularly soft robotic gloves, have significant potential to improve patient outcomes. While vision-based algorithms pave the way for fast and convenient hand pose estimation, most current models struggle to accurately track hand movements when soft robotic gloves are used, primarily due to severe occlusion. This limitation reduces the applicability of soft robotic gloves in digital and remote rehabilitation assessment. Furthermore, traditional clinical assessments like the Fugl-Meyer Assessment (FMA) rely on manual measurements and subjective scoring scales, lacking the efficiency and quantitative accuracy needed to monitor hand function recovery in data-driven personalised rehabilitation. Consequently, few integrated evaluation systems provide reliable quantitative assessments. In this work, we propose an RGB-based evaluation system for soft robotic glove applications, which is aimed at bridging these gaps in assessing hand function. By incorporating the Hand Mesh Reconstruction (HaMeR) model fine-tuned with motion capture data, our hand estimation framework overcomes occlusion and enables accurate continuous tracking of hand movements with reduced errors. The resulting functional metrics include conventional clinical benchmarks such as the mean per joint angle error (MPJAE) and range of motion (ROM), providing quantitative, consistent measures of rehabilitation progress and achieving tracking errors lower than 10°. In addition, we introduce adapted benchmarks such as the angle percentage of correct keypoints (APCK), mean per joint angular velocity error (MPJAVE) and angular spectral arc length (SPARC) error to characterise movement stability and smoothness. This extensible and adaptable solution demonstrates the potential of vision-based systems for future clinical and home-based rehabilitation assessment.

## 1. Introduction

Hand function plays a crucial role in daily activities, and loss of hand motor abilities including the joint range of motion (ROM) can significantly impact a person’s quality of life [1]. In hand function rehabilitation, soft robotic gloves and assistive technologies have gained emerging interest for aiding hand function recovery. Soft robotic gloves are wearable assistive devices with compliant, pneumatically or tendon-driven actuators that support finger flexion and extension during rehabilitation tasks, enabling safe, adaptive and user-comfortable assistance for motor recovery [2,3,4,5]. While recent advances in computer vision have shown excellent results in bare-hand posture estimation, applying these models to evaluate hand function with soft robotic gloves remains challenging [6,7]. The primary difficulty lies in accurately tracking hand poses when the patient’s hand is fully or partially covered by soft robotic gloves. Issues like occlusion, finger slippage and glove size variability also contribute to significant tracking errors [8,9,10]. When parts of the hand or fingers are hidden from the camera’s view, current vision-based models struggle to maintain accuracy [11,12,13,14].

Moreover, an effective approach to quantitatively evaluate hand function as well as the effectiveness of the latest technologies, including soft robotic gloves, remains an open challenge. Clinically, the ROM is often assessed through structured tests like the Fugl-Meyer Assessment (FMA) [15]. These standardised tests evaluate the hand motor function, balance and sensation of patients by scoring their performance in various movements across multiple joints and fingers as well as in both hands. However, they typically rely on manual measurements and subject scoring scales, which can yield inconsistent results and demand considerable staff time, thereby limiting their usability. Quantitative metrics like the joint ROM in degrees provide more precise information to help clinicians understand patient recovery stages and further develop personalised recovery plans.

Recent advances have explored embedding sensors in soft robotic gloves to quantitatively measure joint movements [16]. For example, flexible sensors and inertial measurement units (IMUs) can provide real-time data on finger joint angles, offering valuable information for feedback control of gloves in rehabilitation [17]. Nevertheless, incorporating embedded sensors would significantly increase the cost of soft robotic gloves, and many flexible sensors still face challenges with reliability and durability under repeated use [16,18]. In contrast, using camera-based systems to measure the posture of soft robotic gloves and evaluate rehabilitation performance presents a promising solution for fast, reliable and low-cost implementation in both clinical and home environments [19,20].

**Figure 1:**
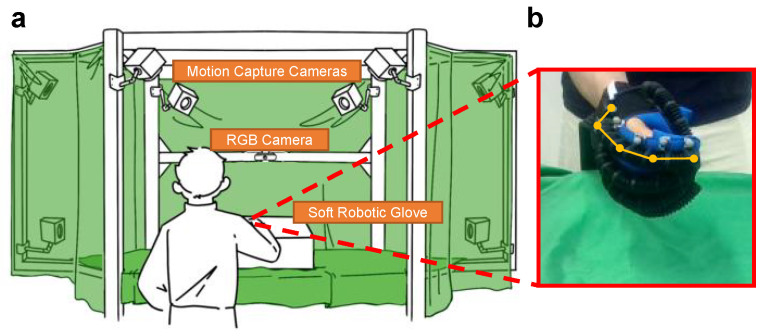
Motion capture system for calibration and visualisation of finger motion with soft robotic gloves. (**a**) Motion capture system. (**b**) Estimated finger motion with the proposed RBG-based model.

The motivation for this research lies in overcoming these limitations. We propose a hand motion evaluation system integrating camera-based hand tracking with minimal fine-tuning of the Hand Mesh Reconstruction (HaMeR) model [7] to manage occlusions caused by gloves or hand positioning, as shown in Figure 1. It compensates for occluded areas, ensuring continuous tracking even in difficult scenarios. Additionally, this method introduces quantitative evaluation metrics, including the range of motion (ROM) and mean per joint angle error (MPJAE), which provide standardised assessments in rehabilitation and clearer insights into movement dynamics. Unlike models that require frequent recalibration, this method offers an adaptable and scalable solution for various rehabilitation applications, achieving tracking errors below 10° even under challenging conditions. The system reduces the need for extensive retraining, making it suitable for long-term rehabilitation in both clinical and home-based settings.

## 2. Materials and Methods

### 2.1. Overview of the Vision-Based Evaluation System

This computer vision-based system was constructed to quantitatively evaluate the hand recovery process with the assistance of soft robotic gloves. We aimed to fine-tune a powerful hand pose estimation model for our scenarios where hands are wearing rehabilitation gloves as well as define the metrics for evaluation.

We applied the HaMeR [7] model for fine-tuning, which is a fully transformer-based approach designed for reconstructing 3D hand meshes from monocular images or video frames. Its architecture leverages a large-scale vision transformer (ViT) as the backbone (also known as H-ViT), which processes the image patches and returns a series of output tokens. It also includes a transformer head to transfer the tokens into MANO and camera parameters, which can then be transformed to joint positions and hand meshes [21]. HaMeR excels in capturing complex hand configurations, thanks to its capacity to scale with larger datasets and utilise powerful deep learning architectures. It consistently outperforms previous state-of-the-art methods in hand pose benchmarks, particularly in challenging wild scenarios, such as hands interacting with objects or other hands, or hands captured from different viewpoints. However, this model still could not be applied to our situation due to the lack of training data captured from hands with rehabilitation gloves, as the testing results turn out to be unsatisfactory in Section 3. Thus, a fine-tuning method is proposed in our system to help bridge this gap in performance.

In evaluation, different from qualitative measurements in clinical tests, we naturally applied the join angle accuracy from hand pose estimation tasks to our framework, including the angle error and the percentage of correctly predicted joints within different error thresholds. These are commonly used metrics for hand pose estimation, especially when joint angles are taken into consideration, from which we could easily calculate the ROM for each joint, which is a comparatively accurate standard of measurement.

**Figure 2:**
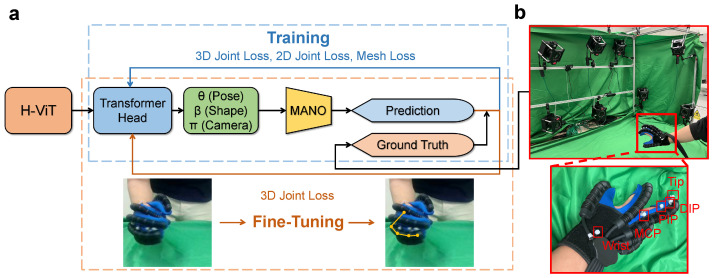
Our training and fine-tuning framework, as well as the data collection system and markers on the glove. The HaMeR model was firstly trained on a large-scale dataset and then fine-tuned on our glove dataset. In data collection, the motion capture system had 8 motion capture cameras to record joint coordinates and 1 RGB camera to capture RGB images. (**a**) Training and fine-tuning framework. (**b**) Data collection system and markers on the glove.

Our training and fine-tuning framework is shown in Figure 2. Firstly, the model was trained following the process from the original HaMeR [7], computing the 3D joint loss, 2D joint loss and mesh loss. After training on large datasets, the transformer head was then fine-tuned on our small-scale dataset collected from gloved hands to fit to our environment, merely considering the 3D joint loss. The details will be explicated in Section 2.3.

### 2.2. Data Collection

As our dataset included RGB images and corresponding 3D joint positions for calibration, we used a motion capture system to capture the data we needed, as shown in Figure 2. Here, we abbreviate the joints of a finger as the metacarpophalangeal joint (MCP), proximal interphalangeal joint (PIP) and distal interphalangeal joint (DIP). Note that the joints of the thumb, from the wrist to the tip, are abbreviated as the trapeziometacarpal joint (TM), MCP and interphalangeal joint (IP). For clarity, the thumb joints (TM–MCP–IP) follow a similar notation scheme to the remaining fingers (MCP–PIP–DIP), and the same abbreviations are used interchangeably when no ambiguity arises. Covered by green cloth, the system used 8 NOKOV MARS4H motion capture cameras (NOKOV Science & Technology Co., Ltd., Beijing, China) to locate the joint coordinates and 1 RGB camera (720 p, 30 fps) facing towards the hand to capture images. To represent the positions of the joints, we used grey spherical markers stuck on the glove. Considering the mutual inference of the markers when all 21 joints of the hands were simultaneously present, in each image, only one finger and the wrist were focused on. Thus, there were five markers to capture.

Our dataset consisted of ∼3000 images for training, ∼1000 for validation and ∼1000 for testing, with corresponding 3D joint positions, captured from one subject. However, current automatic 2D hand pose annotation methods such as MMPose [22] have been proven to perform poorly, especially when dealing with hand poses with self- and object occlusions. That aside, manually annotating 2D keypoints for each view is fairly expensive for data collection. However, our experiments have shown that 3D data alone is enough for fine-tuning. In addition, mesh data requiring scanners to collect is also unnecessary in our framework.

### 2.3. Training and Fine-Tuning

In the training process, the dataset was contributed by the authors of HaMeR [7], which consists of 40,400 RGB images in total. The model computes the 3D joint position losses, 2D joint position losses and mesh losses. For the ground-truth 3D joint positions $X^{*}$, 2D joint positions $x^{*}$ and MANO parameters $\theta^{*},\beta^{*}$ and the predicted 3D joint positions *X*, 2D joint positions *x* and MANO parameters θ,β, the loss is calculated as follows [7]:

(1) $$\begin{array}{cc} L_{Training} & =w_{3D}L_{3D}+w_{2D}L_{2D}+w_{MANO}L_{MANO}\\ \; & =w_{3D}||X-X^{*}\left|\right|+w_{2D}||x-x^{*}\left|\right|+\\ \; & w_{MANO}(||\theta -\theta^{*}\left|\right|+||\beta -\beta^{*}\left||\right) \end{array}$$

where $w_{3D},w_{2D},w_{MANO}$ refer to the weights of the 3D (0.05), 2D (0.01) and MANO parameter (0.0005) losses, respectively. Different from the training process, we merely utilised the 3D joint losses for fine-tuning:

(2) $$L_{Fine-Tuning}=L_{3D}=||X-X^{*}\left|\right|$$

This simplified process gets rid of complex annotation of the 2D ground truth joint positions and supervises the level of the 3D joints, encouraging consistency in the 3D space.

### 2.4. Implementation Details

All the experiments were conducted on a single Tesla V100 GPU, with Python 3.10.15, PyTorch 2.6.0 + CUDA 12.6 on a Linux workstation. Numerical and scientific computations were performed with NumPy 1.26.1 and SciPy 1.14.1, while image processing and visualisation relied on OpenCV 4.11.0 and Matplotlib 3.9.2. The MANO hand model used in this work corresponds to version 1.2, obtained from the official release [21]. All software packages were installed in a controlled environment to ensure reproducibility.

In training, all the settings remained the same as those provided by the authors of HaMeR [7], with the number of epochs being 1000, the learning rate being $1\times 10^{-5}$ and the weight decay being $1\times 10^{-4}$ In the fine-tuning process, the number of epochs was 40, with a learning rate of $1\times 10^{-5}$ and weight decay of $1\times 10^{-4}$. Aside from that, as in our data collection, the markers were stuck on the glove rather than the skin, and there was an offset between the real joint positions and those of the markers. As the offset of the markers on the fingers was the same, the model could adapt to the offset during the fine-tuning process. However, the marker representing the wrist was comparatively far from the ground truth, and thus we added 40° to the output MCP angle (since the marker was a bit higher than the skin). Note that this did not influence the fine-tuning process, as the angles were calculated from the joint positions and not included in the loss computation. We compare the performance of our fine-tuned HaMeR model with the model without fine-tuning as the baseline to prove the effect of our framework.

## 3. Results

In addition to the original HaMeR model, we included Hamba as a representative interaction-aware RGB-based baseline [23]. Hamba has demonstrated robustness in challenging hand–object interaction scenarios and serves as a competitive comparison for evaluating performance under glove-induced occlusion.

### 3.1. Joint Angle Accuracy

To evaluate the accuracy of three joint angles per finger, we chose the mean per joint angle error (MPJAE) [24] to measure the error:

(3) $$MPJAE=\frac{1}{N}\sum_{i=1}^{N}\left|\theta_{i}-\theta_{i}^{*}\right|$$

where *N* is the number of joints and $\theta_{i}^{*}$ and $\theta_{i}$ are the ground-truth and predicted angles, respectively. Based on the percentage of correct keypoints (PCK) score [25], we introduce the angle PCK (APCK) score, which represents the percentage of correctly predicted angles with an error threshold (unit: °). Subsequently, we could draw the curve of the APCK score with different thresholds and calculate the area under the curve (AUC).

**Figure 3:**
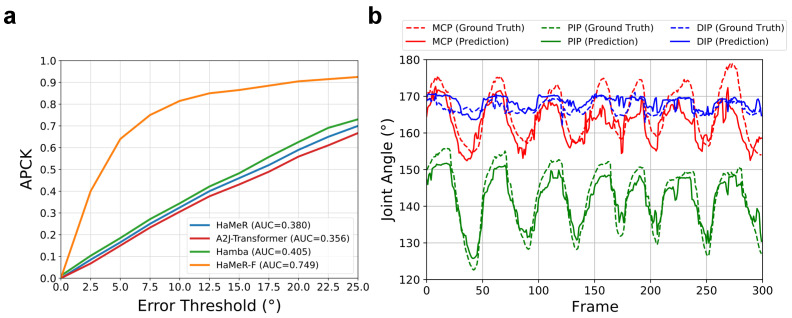
(**a**) APCK curves of HaMeR model and our fine-tuned model. The score of our model was apparently higher than that of the original HaMeR model. (**b**) Joint angle curves for the middle finger. Our model successfully achieved the temporal robustness for predicting joint angles, with errors lower than 10°.

We present the MPJAE and angle PCK scores with error thresholds of 5° and 10° in Table 1, where we abbreviate our fine-tuned HaMeR model as HaMeR-F. We observed that over 60% of the angle prediction reached an error lower than 5°, and more than 80% of the angles were accurately predicted with a threshold of 10°. The APCK score curves with different error thresholds are illustrated in Figure 3a, where the AUC of our fine-tuned model reached almost twice those of the Hamba model and HaMeR model without fine-tuning. However, it can also be seen that the model performed worst for the MCP angle. This phenomenon can be partly explained by the huge offset of the wrist, which is hard to concisely rectify in different environments and with different movements.

We also demonstrate the ground-truth and predicted ROMs of the joints in Table 2, each presented as the minimum and maximum angle of the joint. That aside, to observe the dynamic estimation of the joint angles, we tested our model on a 10 s (300 frames) video recording movements of the middle finger and compared the predicted angle curves with the ground truth in Figure 3b. In general, the output results manifested the finger movements in the temporal sequence well, further validating its feasibility in ROM monitoring.

### 3.2. Qualitative Results

**Figure 4:**
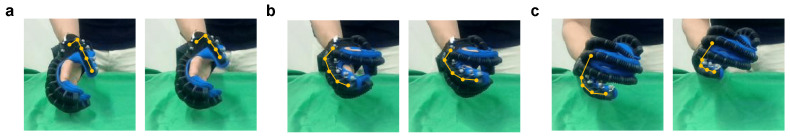
Qualitative results of predictions. Our model accurately estimated finger poses at different angles. (**a**) Thumb motions. (**b**) Middle finger motions. (**c**) Pinky finger motions.

We demonstrate here the visualisation of 3D hand joints for the thumb, middle finger and pinky finger in Figure 4. After fine-tuning, the model became consistently robust and accurate in predicting the joint angles as well as the finger poses, even with the glove.

### 3.3. Ablation Study: 3D Joint Accuracy and Kinematic Smoothness

In this ablation study, we further analysed both the 3D joint accuracy and the kinematic quality of the reconstructed motion. The 3D accuracy was quantified with the mean per joint position error MPJPE, which measures the Euclidean distance between the predicted and ground-truth joint positions in millimetres. Beyond static positional accuracy, we introduce two kinematic-level metrics to assess temporal fidelity:

- Mean per Joint Angular Velocity Error MPJAVE This was computed as the mean absolute difference between the predicted and ground-truth angular velocities (in °/s) of each joint. This metric reflects how well the model captured the motion dynamics, i.e., the speed consistency of each finger joint during flexion and extension. A smaller MPJAVE indicates more temporally stable motion estimation.
- Angular SPARC Error: This is derived from the spectral arc length SPARC metric, a frequency-domain measure of motion smoothness [26]. The SPARC quantifies how smoothly a joint angle trajectory evolves over time by integrating the curvature of its amplitude spectrum. Here, we report the absolute SPARC difference between the estimated and ground-truth trajectories, where lower values denote higher smoothness consistency.

Table 3 summarises the per-joint results of the baseline models (HaMeR and Hamba) and the fine-tuned model (HaMeR-F). While fine-tuning significantly improved the spatial accuracy in terms of joint position and angle estimation, the gains in temporal metrics such as the MPJAVE and SPARC were more limited. This is expected, as the fine-tuning process optimises the per-frame spatial alignment using 3D joint supervision without explicitly modeling temporal dynamics or motion smoothness priors. Consequently, improvements in velocity consistency and spectral smoothness arise primarily as indirect effects of reduced spatial noise rather than from dedicated temporal constraints. Incorporating sequence-level supervision or explicit temporal regularisation is therefore a promising direction for further improving kinematic smoothness.

## 4. Discussion

The paper propels the use of soft-robotic-glove-based hand and finger tracking into more accessible rehabilitation environments, particularly by improving its integration into clinical settings. With the fine-tuned vision-based model and the use of an RGB-based camera, this approach offers a promising solution for estimating the ROMs for hands accurately. This method also offers standardised assessments for rehabilitation by building quantitative evaluation metrics, such as the ROM, MPJAE, MPJPE, MPJAVE and SPARC errors, which provide clearer insights into movement dynamics.

However, it should be noted that the current study focused on a subject-specific calibration scenario, with all training, validation and testing data collected from a single participant wearing one soft robotic glove under controlled conditions. This setting is aligned with practical rehabilitation use cases, where soft robotic gloves are typically calibrated to individual users due to variations in hand size, glove fit and motor ability. With calibration, the proposed framework already enables accurate and continuous estimation of joint angles and functional metrics (e.g., ROM and kinematic consistency) using only an RGB camera, providing a low-cost and easily deployable evaluation tool that can be readily adopted for subject-level assessment, system benchmarking and algorithm validation.

At the same time, cross-subject generalisation remains a well-recognised challenge in vision-based hand pose estimation. Prior studies have shown that models trained on limited subject populations often struggle to transfer reliably to unseen individuals with different hand geometries, appearance characteristics and motion patterns, particularly under occlusion and domain shifts [27]. Recent systematic reviews further indicate that despite rapid progress in deep learning-based methods, robust generalisation across diverse real-world conditions and subject populations is still an open problem due to anatomical variability, occlusions and dataset bias [28]. Thus, the present study is positioned as an initial feasibility investigation rather than a cross-subject evaluation.

The evaluation was therefore conducted using isolated single-finger motions, fixed camera viewpoints and uniform backgrounds to intentionally isolate the effect of visual occlusion in a controlled setting. In addition, the wrist angle correction applied in this study was used only during post-processing for evaluation purposes and did not affect model training or optimisation. This subject-specific adjustment further motivates the need for systematic calibration strategies. Building on the current framework, future work will expand the dataset to include multiple subjects and more diverse motion patterns, investigate cross-subject generalisation strategies and explore more comprehensive functional and kinematic metrics that better capture movement coordination, temporal consistency and rehabilitation-relevant performance beyond joint-level accuracy.

Although clinical relevance is a central motivation of this work, therapist involvement in the present study was limited and did not include qualitative clinical assessments. Consequently, the study did not include validation of patient populations or direct alignment with established clinical assessment scales, such as the Fugl-Meyer Assessment (FMA) scale [15]. The quantitative metrics reported here (ROM, MPJAE, MPJPE, MPJAVE and angular SPARC error) are therefore intended as complementary, objective descriptors rather than replacements for clinician-rated scales. From a clinical workflow perspective, the proposed system is best regarded as a supportive tool for assessments with subject-specific calibration, enabling quantitative monitoring of rehabilitation progress alongside established protocols. There will also be limitations when extending the approach to pathological movement patterns such as spasticity, abnormal synergies or compensatory motions, which will require pathology-specific data collection, temporal modeling and therapist-guided validation. Future work will focus on clinical studies to relate the proposed quantitative metrics to established clinical assessments and to facilitate translation from laboratory evaluation to real-world rehabilitation practice.

## Figures and Tables

**Table 1:** 3D joint angle estimation.

Joint	Method	MPJAE (°)	APCK@5	APCK@10
MCP	HaMeR	29.76 ± 17.78	0.071	0.126
	Hamba	25.62 ± 15.61	0.082	0.137
	HaMeR-F	9.39 ± 8.58	0.288	0.514
PIP	HaMeR	15.44 ± 10.30	0.176	0.361
	Hamba	12.28 ± 11.76	0.183	0.376
	HaMeR-F	3.32 ± 2.89	0.783	0.957
DIP	HaMeR	12.85 ± 9.97	0.263	0.493
	Hamba	10.67 ± 13.58	0.290	0.519
	HaMeR-F	2.74 ± 2.83	0.861	0.969
Overall	HaMeR	19.35 ± 15.14	0.170	0.326
	Hamba	16.19 ± 15.98	0.185	0.344
	HaMeR-F	5.48 ± 6.17	0.644	0.814

**Table 2:** 3D joint ROM evaluation (°).

Finger	Result	MCP	PIP	DIP
Thumb	GT	100.6∼108.7	130.0∼174.5	143.8∼179.4
	HaMeR-F	100.0∼109.6	131.2∼170.2	151.9∼176.5
Index	GT	155.9∼176.4	120.4∼152.9	154.8∼172.4
	HaMeR-F	150.3∼169.4	126.8∼151.0	151.8∼169.0
Middle	GT	148.0∼179.3	123.7∼155.7	164.4∼172.1
	HaMeR-F	140.0∼169.3	129.7∼148.1	161.1∼169.0
Ring	GT	126.3∼157.5	143.2∼150.9	158.8∼174.1
	HaMeR-F	120.8∼159.6	145.0∼153.9	158.0∼165.3
Pinky	GT	150.0∼163.4	129.8∼151.5	159.2∼172.7
	HaMeR-F	140.0∼159.3	137.6∼152.0	158.0∼167.0

**Table 3:** 3D joint position and kinematic accuracy evaluation, where the MPJAVE and Angular SPARC Error for the wrist and tip cannot be meaningfully computed due to the lack of well-defined flexion–extension trajectories at these joints, and are therefore marked as N/A.

Joint	Method	MPJPE (mm)	MPJAVE (°/s)	Angular SPARC Error
Wrist	HaMeR	24.02 ± 5.68	N/A	N/A
	Hamba	23.09 ± 4.77	N/A	N/A
	HaMeR-F	23.06 ± 2.00	N/A	N/A
MCP	HaMeR	15.85 ± 3.71	40.49 ± 8.94	2.32 ± 2.29
	Hamba	14.37 ± 3.68	38.95 ± 8.68	2.20 ± 2.27
	HaMeR-F	12.15 ± 1.57	30.47 ± 4.99	1.73 ± 2.25
PIP	HaMeR	15.52 ± 3.45	78.98 ± 8.70	3.55 ± 1.22
	Hamba	15.20 ± 3.38	75.23 ± 8.56	3.39 ± 1.20
	HaMeR-F	12.23 ± 1.63	63.71 ± 7.52	2.90 ± 0.96
DIP	HaMeR	14.88 ± 3.35	62.12 ± 9.04	2.04 ± 2.05
	Hamba	14.22 ± 2.99	58.98 ± 8.87	1.92 ± 1.95
	HaMeR-F	12.32 ± 1.74	46.78 ± 5.38	1.28 ± 1.73
Tip	HaMeR	14.28 ± 3.31	N/A	N/A
	Hamba	13.96 ± 3.20	N/A	N/A
	HaMeR-F	12.40 ± 1.86	N/A	N/A
Overall	HaMeR	16.91 ± 4.39	60.53 ± 8.89	2.64 ± 1.91
	Hamba	16.17 ± 3.74	57.72 ± 8.27	2.50 ± 1.85
	HaMeR-F	14.43 ± 2.24	46.99 ± 6.06	1.97 ± 1.73

## Data Availability

Data are available upon request from the corresponding author.

## Abbreviations

The following abbreviations are used in this manuscript:

HaMeR	Hand mesh reconstruction
ROM	Range of motion
MPJAE	Mean per joint angle error
MPJAVE	Mean per joint angular velocity error
FMA	Fugl-Meyer Assessment
MMIB	Mesh-Mano interaction block
CNN	Convolutional neural network
GCN	Graph convolutional network
MCP	Metacarpophalangeal joint
PIP	Proximal interphalangeal joint
DIP	Distal interphalangeal joint
TM	Trapeziometacarpal joint
IP	Interphalangeal joint
PCK	Percentage of correct keypoints
APCK	Angle percentage of correct keypoints
AUC	Area under the curve
SPARC	Spectral arc length
